# Stent Selection for Primary Angioplasty and Outcomes in the Era of Potent Antiplatelets. Data from the Multicenter Randomized Prague-18 Trial

**DOI:** 10.3390/jcm10215103

**Published:** 2021-10-30

**Authors:** Ota Hlinomaz, Zuzana Motovska, Jiri Knot, Roman Miklik, Mahmoud Sabbah, Milan Hromadka, Ivo Varvarovsky, Jaroslav Dusek, Michal Svoboda, Frantisek Tousek, Bohumil Majtan, Stanislav Simek, Marian Branny, Jiří Jarkovský

**Affiliations:** 1 ICRC, Department of Cardioangiology, St. Anne University Hospital, Masaryk University, 65691 Brno, Czech Republic; ota.hlinomaz@fnusa.cz (O.H.); dr1sabbah@yahoo.com (M.S.); 2 Third Faculty of Medicine, Charles University and University Hospital Kralovske Vinohrady, Cardiocentre, 10034 Prague, Czech Republic; knot@centrum.cz; 3 Department of Internal Medicine and Cardiology, Faculty of Medicine of Masaryk University and University Hospital, 62500 Brno, Czech Republic; romanmiklik@yahoo.com; 4 Faculty of Medicine, Suez Canal University, Ismailia P.O. Box 41522, Egypt; 5 Department of Cardiology, University Hospital and Faculty of Medicine, Charles University, 30599 Pilsen, Czech Republic; hromadka@fnplzen.cz; 6 Cardiology Centre AGEL, 53203 Pardubice, Czech Republic; ivovarvarovsky@gmail.com; 7 First Department of Internal Medicine, University Hospital Hradec Kralove, 50005 Hradec Kralove, Czech Republic; jaroslav.dusek@fnhk.cz; 8 Faculty of Medicine, Institute of Biostatistics and Analyses, Masaryk University, 62500 Brno, Czech Republic; svoboda@iba.muni.cz (M.S.); jarkovsky@iba.muni.cz (J.J.); 9 Cardiocentre—Department of Cardiology, Regional Hospital, 37001 Ceske Budejovice, Czech Republic; tousek@nemcb.cz; 10 Cardiocentre, Regional Hospital, 36001 Karlovy Vary, Czech Republic; bohumil@majtan.cz; 11 Department of Physiology and Second Department of Medicine—Department of Cardiovascular Medicine, 1st Faculty of Medicine, Charles University, 12808 Prague, Czech Republic; ssime@lf1.cuni.cz; 12 Cardiovascular Center, Hospital Podlesi, AGEL Research and Training Institute, 73961 Trinec, Czech Republic; marian.branny@fno.cz

**Keywords:** acute myocardial infarction, primary angioplasty, drug-eluting stent, bare-metal stent, bioresorbable scaffolds, ticagrelor, prasugrel

## Abstract

Drug-eluting stents (DES) are the recommended stents for primary percutaneous coronary intervention (PCI). This study aimed to determine why interventional cardiologists used non-DES and how it influenced patient prognoses. The efficacy and safety outcomes of the different stents were also compared in patients treated with either prasugrel or ticagrelor. Of the PRAGUE-18 study patients, 749 (67.4%) were treated with DES, 296 (26.6%) with bare-metal stents (BMS), and 66 (5.9%) with bioabsorbable vascular scaffold/stents (BVS) between 2013 and 2016. Cardiogenic shock at presentation, left main coronary artery disease, especially as the culprit lesion, and right coronary artery stenosis were the reasons for selecting a BMS. The incidence of the primary composite net-clinical endpoint (EP) (death, nonfatal myocardial infarction, stroke, serious bleeding, or revascularization) at seven days was 2.5% vs. 6.3% and 3.0% in the DES, vs. with BMS and BVS, respectively (HR 2.7; 95% CI 1.419–5.15, *p* = 0.002 for BMS vs. DES and 1.25 (0.29–5.39) *p* = 0.76 for BVS vs. DES). Patients with BMS were at higher risk of death at 30 days (HR 2.20; 95% CI 1.01–4.76; for BMS vs. DES, *p* = 0.045) and at one year (HR 2.1; 95% CI 1.19–3.69; *p* = 0.01); they also had a higher composite of cardiac death, reinfarction, and stroke (HR 1.66; 95% CI 1.0–2.74; p = 0.047) at one year. BMS were associated with a significantly higher rate of primary EP whether treated with prasugrel or ticagrelor. In conclusion, patients with the highest initial risk profile were preferably treated with BMS over BVS. BMS were associated with a significantly higher rate of cardiovascular events whether treated with prasugrel or ticagrelor.

## 1. Introduction

The current generation of drug-eluting stents (DES) has been shown to be superior to bare-metal stents (BMS) in reducing the risk of recurrent myocardial infarction (MI), stent thrombosis, and target lesion revascularization [1,2]. Therefore, the guidelines preferentially recommend DES in the context of acute myocardial infarction (AMI) (Class I, Level of Evidence A) [3,4]. The use of bioresorbable vascular scaffolds (BVS) has been hypothesized to overcome the limitations of DES due to their restoration of native vessel physiological motion in the long term. Several randomized clinical trials (RCTs) have compared the safety, clinical and angiographic efficacy, and healing response of BVSs to 2nd generation DES in patients undergoing percutaneous coronary intervention (PCI) for AMI [5,6]. They showed non-inferiority in early clinical and angiographic outcomes and comparable arterial healing; nonetheless, there were safety concerns related to increased rates of composite device-related adverse events and device thrombosis [7]. Therefore, the Task Force on Myocardial Revascularization of the European Society of Cardiology recommended that BVS should not be used outside well-controlled clinical studies [4].

There are several important factors that influence stent choice, e.g., scientific knowledge based on major clinical trial results, the surgeon’s experience with different types of stents, stent availability, cost, and reimbursement. Our study sought to determine (1) the reasons why different types of stents were used in AMI patients, randomized to the PRAGUE-18 study, who underwent primary angioplasty, and (2) how it influenced the prognoses of the study population. The efficacy and safety outcomes of the different stent types were also compared in patients treated with prasugrel vs. ticagrelor.

## 2. Materials and Methods

The multicenter PRAGUE-18 study was an academic open-label phase IV RCT that compared the safety and efficacy of prasugrel vs. ticagrelor in AMI patients treated with primary PCI (pPCI). A detailed study protocol and methodology have already been published [8]. The indication for PCI after coronary angiography and the procedural details, including choice of stent type, length, and size, were not influenced by study protocol and were left to the discretion of the treating interventional cardiologist, as was the decision to administer any adjunctive medication in support of PCI. In patients treated with BVS, pre-dilation was strongly encouraged. However, the implantation technique was at the surgeon’s discretion, and there was no explicit provision for the methods to be used for vessel and device sizing or for post-dilation. All patients were instructed to follow all guideline recommended medications throughout the study period. The primary composite net-clinical endpoint consisted of all-cause death, non-fatal myocardial infarction, stroke, serious bleeding requiring transfusion or prolonging hospitalization, or urgent target vessel revascularization within 7 days after randomization or at discharge, if prior to the 7th day. Clinical follow-up was done on the 7th day or at hospital discharge, whichever came first, and at 30 days and 12 months.

The study design and protocol were approved by the Ethics Committee for Multicenter Clinical Trials, University Hospital Kralovske Vinohrady, Prague, Czech Republic, and the local ethics committees at each participating site. The study protocol was registered under PRAGUE-18 Clinicaltrials.gov NCT02808767. All patients signed informed consent before the intervention [8,9]. 

### 2.1. Study Patients

Between April 2013 and May 2016, 1230 patients were enrolled in the PRAGUE-18 study at 14 sites across the Czech Republic. A total of 1151 stents were implanted. We only analyze patients in whom one type of stent was used (*n* = 1111). Patients receiving more than one type of stent (*n* = 40) were excluded from the analysis. DES were implanted in 749 patients (67.4%), BMS in 296 (26.6%), and BVS in 66 (5.9%) patients. Patients with DES were compared to those with BMS and BVS relative to baseline demographics and procedural characteristics as well as the in-hospital, 30-day, and 365-day endpoint occurrences. The efficacy and safety outcomes of the different stent types (DES vs. BMS vs. BVS) were also compared relative to the study medication (prasugrel vs. ticagrelor). Since 586 (52.7%) patients discontinued the study medication during the trial (mostly for economic reasons), the results for the different stent groups at one year must be considered biased, and we present them as a curiosity.

### 2.2. Statistical Analysis

Data are presented as median, supplemented by the 5th–95th percentile range or counts (%). Categorical variables were compared between treatment groups using Fisher’s exact test; continuous variables were compared using the Kruskal-Wallis test. Survival analysis was done using the Kaplan–Meier methodology. Hazard ratios (HRs) and *p*-values for treatment effects were calculated using Cox proportional hazards models. A *p*-value of < 0.05 was considered statistically significant. All analyses were performed using SPSS version 24.0.0.1 (IBM Corporation, Armonk, New York, NY, USA).

## 3. Results

### 3.1. Baseline Patient Characteristics

Table 1a,b shows the comparison of baseline clinical and procedural features between patient groups relative to implanted stent type (DES vs. BMS vs. BVS). Compared to patients with DES or BMS, patients with BVS were younger, less obese, had lower body mass indexes (BMI), were more often smokers, and had significantly lower levels of urea and creatinine. We did not find any difference in the presence of hyperlipidemia, hypertension, diabetes mellitus or chronic renal failure between DES and BMS patients. 

Patients with BMS implants were more likely to have an AMI with an LBBB morphology on the initial ECG (3.4% vs. 0.9% vs. 1.5% in DES and BVS, respectively, *p* = 0.019). BMS were less likely to be implanted in left anterior descending (LAD) lesions compared with DES (19.2% vs. 74.3%, *p* < 0.001) and more likely to be implanted in right coronary artery (RCA) lesions (31.0% vs. 62.9%, *p* = 0.018). BVS were never implanted in AMI patients who presented with severe heart failure/cardiogenic shock (Killip III-IV) or patients with a left main culprit lesion. On the other hand, BMS were often used in patients in cardiogenic shock (48.8%) or with left main stenosis (47.1%), especially when it was also the culprit lesion (63.6%).

### 3.2. Endpoint Occurrence in Relation to Stent Type

All patients completed the 12-month follow-up after enrollment. The primary net-clinical endpoint (i.e., death, nonfatal MI, stroke, major bleeding, and revascularization) at 7 days was 2.5%, 6.3%, and 3.0% for DES, BMS, and BVS, respectively, with a hazard ratio (HR) of 2.70; 95% confidence interval (CI) 1.42 to 5.15, *p* = 0.002 for BMS vs. DES, and HR 1.25; CI 0.29 to 5.39, *p* = 0.763 for BVS vs. DES (Table 2).

**Figure 1:**
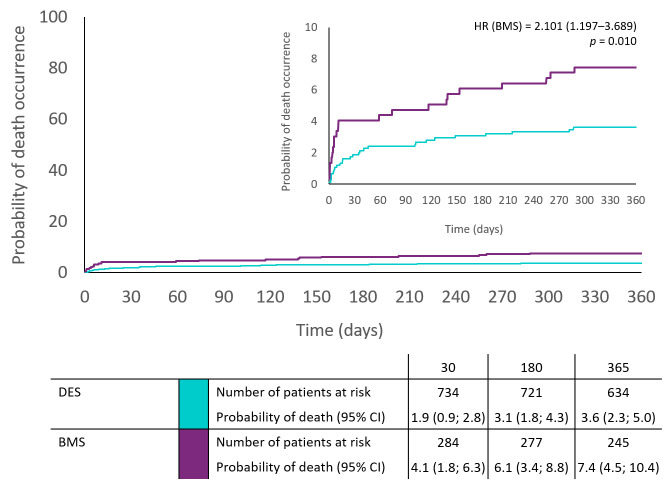
Kaplan-Meier curves of cumulative incidence of death during 365 days in DES and BMS group.

Concerning the occurrence of secondary endpoints at 30 days, patients with BMS were more likely to have higher death rates compared to those with DES (HR 2.20; 95% CI: 1.02 to 4.76; *p* = 0.045). There were no significant differences between groups in cardiovascular death, myocardial reinfarction, stent thrombosis, and bleeding at 30 days. Figure 1 shows the cumulative incidence of the death rate at 365 days, estimated using Kaplan-Meier curves for the DES (Blue) and BMS (Red). Patients with BMS were more likely to have a higher risk of death (HR: 2.1; 95% CI 1.19 to 3.69; *p* = 0.010) and a higher composite of cardiac death, re-MI, and stroke (HR: 1.66; 95% CI: 1.0 to 2.74; *p* = 0.047), compared to those with DES (Table 2).

Compared to DES, the rate of confirmed stent thrombosis in BVS was comparable at 30 days (1.5% vs. 0.8%, HR: 1.89; 95% CI: 0.22 to 15.75; *p* = 0.553), and at 365 days (1.5% vs. 1.3%, HR: 1.13; 95% CI: 0.14 to 8.82; *p* = 0.907) (Table 2). The rate of stent thrombosis in BMS compared to DES was not statistically different (0.7% vs. 0.8%, HR: 0.84; 95% CI: 0.17 to 4.19; *p* = 0.838) at 30 days, and (1.0% vs. 1.3%, HR: 0.77; 95% CI: 0.21 to 2.79; *p* = 0.690) at 365 days.

### 3.3. Endpoint Occurrence in Relation to Stent Type in Patients Treated with Prasugrel vs. Ticagrelor

The incidence of the primary net-clinical endpoint among prasugrel-treated patients was 2.6% in DES patients, 6.3% in BMS (HR 2.74; 95% CI 1.09 to 6.92; *p* = 0.032), and 4.7% in BVS (HR 1.98; 95% CI 0.42 to 9.19; *p* = 0.380).

The incidence of the primary net-clinical endpoint on ticagrelor was 2.5% with DES and 6.6% with BMS (HR 2.65; 95% CI 1.07 to 6.52; *p* = 0.034). No recorded events in the BVS group were observed (Table 3).

## 4. Discussion

Bare-metal stents were originally designed to treat major coronary dissections, avoid acute vessel closure, and prevent restenosis. However, due to a 20–30% rate of angiographic restenosis, BMS were called the Achilles’ heel of PCI. Although many efforts were made to reduce restenosis by modifying stent design and materials, reducing the thickness of stent struts has proven to be the only modification capable of reducing BMS restenosis [10]. First-generation DES significantly reduced angiographic restenosis and ischemia-driven target vessel revascularization. While higher rates of late and very late stent thrombosis were found, no significant differences in long-term death or MI after 1^st^ generation DES vs. BMS implants were observed. BVS were a promising concept in the previous decade, but due to higher thrombosis rates, they are now usually only implanted as part of clinical trials. Second-generation DES have proved to be safer and more effective than 1^st^ generation DES, and are therefore the default stent for all patients, irrespective of clinical presentation, lesion subtype, concomitant therapies, and comorbidities [4,11,12]. The use of biodegradable-polymer DES may further improve clinical outcomes in patients with AMI undergoing primary PCI [13,14].

This report analyzes why different types of stents were used in patients with an AMI treated with primary PCI in the PRAGUE-18 study in the Czech Republic between April 2013 and May 2016, and how it influenced their prognosis. Our main findings are as follows: (1) Patients with the highest risk profile were preferentially treated with BMS over BVS, (2) AMI patients who underwent primary PCI with BVS had comparable cardiovascular outcomes to those who received DES, and (3) BMS implants in AMI patients were associated with a significantly higher rate of composite cardiovascular events regardless of whether they were treated with prasugrel or ticagrelor. We had no intention to directly compare DES vs BMS vs BVS and to evaluate the long-term prognosis of patients after different stent implantation.

The results of our analysis are consistent with earlier clinical practice results in the Czech Republic and other countries [15,16,17,18]. The main reasons for using BMS were the presence of large coronary vessels (requiring implantation of a large stent), ST-elevation myocardial infarction, experience of the operator, costs and reimbursement, regulatory reasons, advanced patient age, and uncertainty regarding the duration of dual antiplatelet therapy, mostly due to higher bleeding risk [19]. A brief survey of the use of BMS found that they were used in 19.6% of cases in Australia [20], 20.0% of cases in the United States [18], 8.9% of cases in Japan [21], and 22.8% in the Czech Republic (2016; unpublished data from the Harmony registry) at the time of PRAGUE-18 study. The percentage of BMS in our study was 26.6%.

The finding that there was no difference between DES and BMS patients in the presence of diabetes mellitus was influenced by two factors. First, all patients were treated by primary PCI strategy immediately after admission to the hospital, when the diagnosis of DM was not often known. Secondly, between 2013 and 2016, Czech interventional cardiologists mostly did not consider diabetes mellitus to be a factor that affected their choice of stent. BMS were implanted mostly for coronary stenoses with higher reference vessel diameters where the risk of in-stent restenosis, target vessel failure, and target vessel revascularization were lower. Since larger coronary vessels (like the left main coronary artery) usually correspond to a larger amount of myocardium supplied by the vessel, it is not surprising that BMS were used more often in AMI in patients with cardiogenic shock or an ECG morphology indicative of an LBBB, i.e., patients with worse prognoses. Recent studies observed that stent efficacy and safety endpoints (DES vs. BMS) consistently favor DES irrespective of implanted stent size [22,23]. In our study, DES were reserved predominantly for patients with stenosis of the proximal or mid-left anterior descending artery where restenosis would have the greatest impact on the patient. BMS were more often implanted in patients with left main and right coronary artery stenosis, which is similar to reports from other authors [22,24]. BVS were the most expensive stents and without sufficient efficacy and safety data at that time; as such, they were implanted rarely and mainly indicated for young, less obese patients without signs of heart failure and with good prognoses.

We did not find significant differences in cardiovascular outcomes between DES and BVS at 7, 30, and 365 days despite the fact that BVS patients were less morbid. Former and recent RTCs and meta-analyses have shown worse or equal mid or long term outcomes of BVS compared to 2nd generation DES, especially if implanted in the context of an AMI [25,26,27,28,29,30,31,32]. The rate of early scaffold/stent thrombosis in our study (BVS 1.5% vs. DES 0.8%) was comparable to that seen in other published reports [27,30,33]. We observed that there was a similar thrombosis rate following BVS and DES implants in patients on prasugrel or ticagrelor at 30 days.

The incidence of the primary composite net-clinical endpoint (all-cause death, non-fatal myocardial infarction, stroke, serious bleeding requiring transfusion or prolonging hospitalization, or urgent target vessel revascularization) at seven days was higher for BMS than DES. Patients with BMS had a higher death rate compared to those with DES at 30 days and 365 days and a higher composite of cardiac death, re-MI, and stroke at 365 days. There were no significant differences between any groups relative to the other endpoints, at 30 or 365 days. The incidence of clinical events was comparable between our analysis and other trials [34,35]. A recent network meta-analysis evaluating long-term stent-related adverse events between BMS and DES confirmed the superiority of 2nd generation DES [16,36]. Patients with implanted BMS had more major adverse cardiovascular events, i.e., target vessel failure, all-cause death, cardiac death, myocardial infarction, revascularization, and stent thrombosis, at one year. Our results are consistent with these findings; however, it is worth noting that the BMS patients in the PRAGUE-18 study had noticeably higher risk profiles. Regardless of whether patients were treated with prasugrel or ticagrelor, we observed comparable findings.

### Study Limitations

The current analysis should be interpreted within the context of several limitations: first, the small number of patients in the BVS group prevented any meaningful conclusions regarding outcomes in this group; second, patient outcomes were affected not only by the type of stent selected but also by risk profile; third, results of the 365-day follow-up for ticagrelor vs. prasugrel must be considered biased, because more than half of the patients discontinued the study medication during the trial.

## 5. Conclusions

Patients with the highest initial risk profile were preferably treated with BMS over BVS. BMS were associated with a significantly higher rate of cardiovascular events whether treated with prasugrel or ticagrelor.

## Figures and Tables

**Table 1:** (a) Comparison of basic clinical parameters between patient groups according to stent type (DES vs. BMS vs. BVS). (b) Comparison of procedural parameters between patient groups according to stent type (DES vs. BMS vs. BVS).

**(a)**					
		**Stent**			***p*-Value**
		**DES (*n* = 749)**	**BMS (*n* = 296)**	**BVS (*n* = 66)**	
Type of acute coronary syndrome					
ST elevations		694 (92.7%)	270 (91.2%)	64 (97.0%)	0.287
LBBB		7 (0.9%)	10 (3.4%)	1 (1.5%)	0.019
RBBB		16 (2.1%)	4 (1.4%)	1 (1.5%)	0.858
Without ST elevations		40 (5.3%)	14 (4.7%)	1 (1.5%)	0.452
Basic characteristics					
Gender—male		574 (76.6%)	223 (75.3%)	45 (68.2%)	0.292
Age		61.7 (42.9; 78.1)	62.7 (46.7; 81.5)	56.9 (40.8; 71.9)	<0.001
BMI		27.8 (22.3; 36.1)	28.3 (22.7; 36.3)	26.4 (21.2; 35.9)	0.022
Laboratory results					
Hemoglobin		144.0 (120; 167.0)	144.0 (118.0;170.0)	144.0 (118.0;170.0)	0.510
Urea		5.2 (3.1; 9.0)	5.4 (3.4; 9.7)	4.9 (2.7; 8.4)	0.011
Creatinine		82.0 (55.0; 124.0)	85.0 (54.0; 136.0)	73.0 (47.0; 106.0)	<0.001
Risk factors and comorbidities					
Hyperlipidemia		269 (35.9%)	93 (31.4%)	15 (22.7%)	0.052
Obesity		155 (20.7%)	53 (17.9%)	6 (9.1%)	0.050
Arterial hypertension		369 (49.3%)	164 (55.4%)	33 (50.0%)	0.198
Smoking		485 (64.8%)	179 (60.5%)	52 (78.8%)	0.016
Diabetes mellitus		157 (21.0%)	62 (20.9%)	9 (13.6%)	0.387
Condition after MI		52 (6.9%)	21 (7.1%)	3 (4.5%)	0.824
Condition after PCI		47 (6.3%)	16 (5.4%)	3 (4.5%)	0.840
Condition after CABG		7 (0.9%)	4 (1.4%)	1 (1.5%)	0.506
Chronic heart failure		7 (0.9%)	3 (1.0%)	0 (0.0%)	0.999
Chronic renal failure		8 (1.1%)	6 (2.0%)	0 (0.0%)	0.380
Bleeding		2 (0.3%)	1 (0.3%)	0 (0.0%)	0.999
Peripheral artery disease		29 (3.9%)	4 (1.4%)	1 (1.5%)	0.078
Killip class	1	667 (89.1%)	253 (85.5%)	64 (97.0%)	0.041
	2	50 (6.7%)	19 (6.4%)	2 (3.0%)	
	3	11 (1.5%)	4 (1.4%)	0 (0.0%)	
	4	21 (2.8%)	20 (6.8%)	0 (0.0%)	
**(b)**					
**Parameter**	**Option**	**Stent**			***p*-Value**
		**DES (*n* = 749)**	**BMS (*n* = 296)**	**BVS (*n* = 66)**	
Coronarography and primary PCI					
TIMI flow in culprit artery after pPCI	3	723 (67.3%)	286 (26.6%)	66 (6.1%)	0.378
	<3	26 (72.2%)	10 (27.8%)	0 (0.0%)	
Number of diseased coronary arteries	1	381 (68.6%)	141 (25.4%)	33 (5.9%)	0.643
	>1	368 (66.2%)	155 (27.9%)	33 (5.9%)	
Left main stenosis ≥50%	Yes	17 (50.0%)	16 (47.1%)	1 (2.9%)	0.036
Left main stenosis as culprit lesion	Yes	4 (36.4%)	7 (63.6%)	0 (0.0%)	0.035
LAD	Yes	332 (74.3%)	86 (19.2%)	29 (6.5%)	<0.001
LAD and Diagonal artery	Yes	43 (70.5%)	15 (24.6%)	3 (4.9%)	0.948
LCx	Yes	80 (65.6%)	37 (30.3%)	5 (4.1%)	0.521
LCx and OM	Yes	48 (63.2%)	26 (34.2%)	2 (2.6%)	0.207
RCA	Yes	288 (62.9%)	142 (31.0%)	28 (6.1%)	0.018
Result of pPCI	Optimal	732 (67.7%)	284 (26.3%)	65 (6.0%)	0.257
	Suboptimal or unsuccessful	17 (56.7%)	12 (40.0%)	1 (3.3%)	

Absolute and relative frequencies were used for categorical variables. Statistical significance of differences between patient groups were tested using Fisher’s exact test. Continuous parameters were described by median (5th; 95th percentile), and statistical significance of differences between patient groups were tested using the Kruskal-Wallis test. LBBB—left bundle branch block, RBBB—right bundle branch block, BMI—body mass index. Categorical parameters are expressed as absolute numbers (percentage of total) and compared using Fisher’s exact test. pPCI -primary PCI, LAD—left anterior descending artery, LCx—left circumflex artery, OM—obtuse marginal artery, RCA—right coronary artery.

**Table 2:** Comparison of endpoint occurrence among patient groups by stent type (DES vs. BMS vs. BVS).

	Stent			*p*-Value	BMS *		BVS *	
	DES	BMS	BVS		HR (95% CI)	*p*	HR (95% CI)	*p*
7 days								
PE (Death/Re-MI/Stroke/Severe bleeding/Revasc)	19 (2.5%)	19 (6.3%)	2 (3.0%)	0.011	2.70 (1.42–5.15)	0.002	1.25 (0.29–5.39)	0.763
30 days								
CV death	12 (1.6%)	9 (3.0%)	1 (1.5%)	0.303	1.92 (0.80–4.55)	0.139	0.94 (0.12–7.23)	0.953
Re-MI	9 (1.2%)	3 (1.0%)	1 (1.5%)	0.791	0.85 (0.23–3.14)	0.808	1.26 (0.16–10.01)	0.822
Stroke	2 (0.3%)	1 (0.3%)	0 (0.0%)	0.999	1.27 (0.11–14.10)	0.841	–	–
CV death/Re-MI/Stroke	19 (2.5%)	13 (4.4%)	2 (3.0%)	0.281	1.75 (0.86–3.55)	0.119	1.20 (0.27–5.15)	0.807
Death	14 (1.9%)	12 (4.1%)	1 (1.5%)	0.101	2.20 (1.02–4.76)	0.045	0.81 (0.11–6.13)	0.835
Stent thrombosis	6 (0.8%)	2 (0.7%)	1 (1.5%)	0.587	0.84 (0.17–4.19)	0.838	1.89 (0.22–15.75)	0.553
Bleeding	40 (5.3%)	24 (8.1%)	3 (4.5%)	0.218	1.57 (0.94–2.61)	0.079	0.85 (0.26–2.77)	0.799
TIMI—severe	3 (0.4%)	4 (1.4%)	0 (0.0%)	0.232	3.43 (0.76–15.33)	0.106	–	–
BARC—severe	7 (0.9%)	6 (2.0%)	0 (0.0%)	0.346	2.21 (0.74–6.58)	0.154	–	–
365 days								
CV death	20 (2.7%)	15 (5.1%)	1 (1.5%)	0.119	1.93 (0.98–3.76)	0.054	0.56 (0.07–4.18)	0.573
Re-MI	20 (2.7%)	8 (2.7%)	1 (1.5%)	0.999	1.03 (0.45–2.34)	0.935	0.56 (0.07–4.19)	0.575
Stroke	6 (0.8%)	3 (1.0%)	1 (1.5%)	0.523	1.29 (0.32–5.18)	0.713	1.85 (0.22–15.42)	0.566
CV death/Re-MI/Stroke	39 (5.2%)	25 (8.4%)	3 (4.5%)	0.150	1.66 (1.01–2.74)	0.047	0.86 (0.26–2.80)	0.810
Death	27 (3.6%)	22 (7.4%)	1 (1.5%)	0.018	2.10 (1.19-3.69)	0.010	0.41 (0.05–3.05)	0.388
Stent thrombosis	10 (1.3%)	3 (1.0%)	1 (1.5%)	0.812	0.77 (0.21–2.79)	0.690	1.13 (0.14–8.82)	0.907
Bleeding	78 (10.4%)	32 (10.8%)	10 (15.2%)	0.461	1.08 (0.71–1.62)	0.715	1.45 (0.75–2.80)	0.268
TIMI—severe	4 (0.5%)	4 (1.4%)	2 (3.0%)	0.051	2.58 (0.64–10.32)	0.180	5.63 (1.03–30.73)	0.046
BARC—severe	12 (1.6%)	6 (2.0%)	2 (3.0%)	0.453	1.29 (0.48–3.44)	0.609	1.87 (0.41–8.36)	0.412

Absolute and relative frequencies were used for categorical variables. Statistical significance of differences between patient groups were tested using the Fisher exact test. The ratio of risk functions is analyzed using the Cox proportional risk model. * Reference Category = DES. HR—hazard ratio, CI—confidence interval, PE—primary endpoint, CV—cardiovascular, Re-MI—myocardial reinfarction, Revasc—revascularization, TIMI—Thrombolysis in Myocardial Infarction, BARC—Bleeding Academic Research Consortium.

**Table 3:** Comparison of endpoint occurrence among patient groups by stent type (DES vs. BMS vs. BVS)—stratified according to study medication (prasugrel and ticagrelor).

	Stent			*p*-Value	BMS *	BVS *		
	DES	BMS	BVS		HR (95% CI)	*p*	HR (95% CI)	*p*
Patients Randomized to Prasugrel								
7 days								
PE (Death/Re-MI/Stroke/Severe bleeding/Revasc)	10 (2.6%)	9 (6.3%)	2 (4.7%)	0.104	2.74 (1.09–6.92)	0.032	1.98 (0.42–9.19)	0.380
30 days								
CV death	6 (1.6%)	5 (3.5%)	0 (0.0%)	0.280	2.30 (0.70–7.55)	0.167	–	–
Re-MI	5 (1.3%)	1 (0.7%)	1 (2.3%)	0.649	0.54 (0.06–4.68)	0.583	1.81(0.21–15.55)	0.586
Stroke	2 (0.5%)	1 (0.7%)	0 (0.0%)	0.999	1.38 (0.12–15.22)	0.792	–	–
CV death/Re-MI/Stroke	11 (2.8%)	7 (4.9%)	1 (2.3%)	0.427	1.75 (0.67–4.51)	0.246	0.82 (0.10–6.39)	0.854
Death	7 (1.8%)	6 (4.2%)	0 (0.0%)	0.203	2.37 (0.79–7.07)	0.120	–	–
In stent thrombosis	2 (0.5%)	1 (0.7%)	1 (2.3%)	0.314	1.36 (0.12–15.08)	0.798	4.53(0.41–50.05)	0.217
Bleeding	23 (5.9%)	10 (7.0%)	3 (7.0%)	0.810	1.22 (0.58–2.56)	0.597	1.20 (0.36–4.00)	0.763
TIMI—severe	2 (0.5%)	2 (1.4%)	0 (0.0%)	0.483	2.77 (0.39–19.73)	0.307	–	–
BARC—severe	5 (1.3%)	2 (1.4%)	0 (0.0%)	0.999	1.11 (0.21–5.73)	0.898	–	–
365 days (biased by high switch rate to clopidogrel)								
CV death	11 (2.8%)	9 (6.3%)	0 (0.0%)	0.081	2.28 (0.94–5.51)	0.066	–	–
Re-MI	12 (3.1%)	3 (2.1%)	1 (2.3%)	0.913	0.69 (0.19–2.46)	0.575	0.74 (0.09–5.70)	0.774
Stroke	4 (1.0%)	2 (1.4%)	1 (2.3%)	0.425	1.40 (0.25–7.67)	0.694	2.19 (0.24–19.59)	0.483
CV death/Re-MI/Stroke	23 (5.9%)	13 (9.2%)	2 (4.7%)	0.398	1.58 (0.80–3.12)	0.186	0.77 (0.18–3.28)	0.728
Death	15 (3.9%)	13 (9.2%)	0 (0.0%)	0.018	2.42 (1.15–5.09)	0.019	–	–
In stent thrombosis	4 (1.0%)	2 (1.4%)	1 (2.3%)	0.425	1.39 (0.25–7.63)	0.699	2.23 (0.25–20.02)	0.471
Bleeding	40 (10.3%)	12 (8.5%)	9 (20.9%)	0.075	0.84 (0.44–1.61)	0.611	2.069 (1.00–4.26)	0.049
TIMI—severe	2 (0.5%)	2 (1.4%)	2 (4.7%)	0.035	2.80 (0.39–19.88)	0.303	8.90 (1.25–63.18)	0.029
BARC—severe	7 (1.8%)	2 (1.4%)	2 (4.7%)	0.325	0.79 (0.16–3.83)	0.777	2.52 (0.52–12.15)	0.248
Patients Randomized to Ticagrelor								
7 days								
PE (Death/Re-MI/Stroke/Severe bleeding/Revasc)	9 (2.5%)	10 (6.6%)	0 (0.0%)	0.080	2.65 (1.07–6.52)	0.034	–	–
30 days								
CV death	6 (1.7%)	4 (2.6%)	1 (4.3%)	0.343	1.58 (0.44–5.60)	0.478	2.61 (0.31–21.68)	0.374
Re-MI	4 (1.1%)	2 (1.3%)	0 (0.0%)	0.999	1.19 (0.21–6.50)	0.839	–	–
Stroke	0 (0.0%)	0 (0.0%)	0 (0.0%)	–	–	–	–	–
CV death/Re-MI/Stroke	8 (2.2%)	6 (3.9%)	1 (4.3%)	0.345	1.78 (0.62–5.15)	0.282	1.95 (0.24–15.66)	0.526
Death	7 (1.9%)	6 (3.9%)	1 (4.3%)	0.265	2.04 (0.68–6.07)	0.199	2.23 (0.27–18.19)	0.451
In stent thrombosis	4 (1.1%)	1 (0.6%)	0 (0.0%)	0.999	0.58 (0.06–5.26)	0.636	–	–
Bleeding	17 (4.7%)	14 (9.1%)	0 (0.0%)	0.090	2.01 (0.99–4.09)	0.052	–	–
TIMI—severe	1 (0.3%)	2 (1.3%)	0 (0.0%)	0.310	4.76 (0.43–52.56)	0.202	–	–
BARC—severe	2 (0.6%)	4 (2.6%)	0 (0.0%)	0.144	4.77 (0.87–26.08)	0.071	–	–
365 days (biased by high switch rate to clopidogrel)								
CV death	9 (2.5%)	6 (3.9%)	1 (4.3%)	0.420	1.58 (0.56–4.44)	0.384	1.74 (0.22–13.79)	0.596
Re-MI	8 (2.2%)	5 (3.2%)	0 (0.0%)	0.742	1.50 (0.49–4.59)	0.475	–	–
Stroke	2 (0.6%)	1 (0.6%)	0 (0.0%)	0.999	1.19 (0.10–13.16)	0.885	–	–
CV death/Re-MI/Stroke	16 (4.4%)	12(7.8%)	1 (4.3%)	0.294	1.80 (0.85–3.80)	0.124	0.98 (0.13–7.38)	0.984
Death	12 (3.3%)	9 (5.8%)	1 (4.3%)	0.315	1.78 (0.75–4.24)	0.188	1.31 (0.17–10.09)	0.794
In stent thrombosis	6 (1.7%)	1 (0.6%)	0 (0.0%)	0.765	0.39 (0.04–3.27)	0.388	–	–
Bleeding	38 (10.5%)	20 (13.0%)	1 (4.3%)	0.496	1.29 (0.75–2.22)	0.351	0.39 (0.05–2.89)	0.363
TIMI—severe	2 (0.6%)	2 (1.3%)	0 (0.0%)	0.653	2.38 (0.33–16.91)	0.385	–	–
BARC—severe	5 (1.4%)	4 (2.6%)	0 (0.0%)	0.638	1.91 (0.51–7.12)	0.333	–	–

Absolute and relative frequencies were used for categorical variables. Statistical significance of differences between patient groups were tested using the Fisher exact test. The ratio of risk functions is analyzed using the Cox proportional risk model. * Reference Category = DES, HR—hazard ratio, CI—confidence interval, PE—primary endpoint, CV—cardiovascular, Re-Mi—myocardial reinfarction, Revasc—revascularization, TIMI—Thrombolysis in Myocardial Infarction, BARC—Bleeding Academic Research Consortium.

## Data Availability

Data available on request due to privacy restrictions.
